# Supplementary material for: Interventions to increase cervical screening uptake among immigrant women: A systematic review and meta-analysis
Source: PLoS One. 2023 Jun 2;18(6):e0281976. doi: 10.1371/journal.pone.0281976 (PMC10237485; doi:10.1371/journal.pone.0281976)
Supplement: S1 File — (PDF) [file pone.0281976.s001.pdf]

Systematic review

Please select one of the options below to edit your record. Either option will create a new version of the record - the existing version will remain unchanged.

A list of fields that can be edited in an update can be found here

1. * Review title. [1 change]

Give the title of the review in English

Effectiveness of interventions for cervical screening uptake among migrant women, a systematic review and meta-analysis

2. Original language title.

For reviews in languages other than English, give the title in the original language. This will be displayed with the English language title.

3. * Anticipated or actual start date.

Give the date the systematic review started or is expected to start.

01/06/2020

4. * Anticipated completion date. [1 change]

Give the date by which the review is expected to be completed.

30/12/2021

5. * Stage of review at time of this submission. [1 change]

This field uses answers to initial screening questions. It cannot be edited until after registration.

Tick the boxes to show which review tasks have been started and which have been completed.

Update this field each time any amendments are made to a published record.

The review has not yet started: No

Review stage	Started	Completed
Preliminary searches	Yes	Yes
Piloting of the study selection process	Yes	Yes
Formal screening of search results against eligibility criteria	Yes	Yes
Data extraction	Yes	Yes
Risk of bias (quality) assessment	Yes	Yes
Data analysis	Yes	No

Provide any other relevant information about the stage of the review here.

While most of the steps in the review stages have been completed as mentioned above, we are working on the qualitative data synthesis and meta-analysis
While most of the steps in the review stages have been completed as mentioned above, we are working on the qualitative data synthesis and meta-analysis

6. * Named contact.

The named contact is the guarantor for the accuracy of the information in the register record. This may be any member of the review team.

Zufishan Alam

Email salutation (e.g. "Dr Smith" or "Joanne") for correspondence:
Dr Alam

7. * Named contact email.

Give the electronic email address of the named contact.

z.alam@uqconnect.edu.au

8. Named contact address

PLEASE NOTE this information will be published in the PROSPERO record so please do not enter private information, i.e. personal home address [1 change]

Give the full institutional/organisational postal address for the named contact.

Centre for Health Services Research,
Level 2, Building 33
Princess Alexandra Hospital campus
Woolloongabba QLD 4102, Australia

9. Named contact phone number. [1 change]

Give the telephone number for the named contact, including international dialling code.

+61478297340

10. * Organisational affiliation of the review.

Full title of the organisational affiliations for this review and website address if available. This field may be completed as 'None' if the review is not affiliated to any organisation.

The University of Queensland

Organisation web address:
www.uq.edu.au

11. * Review team members and their organisational affiliations. [1 change]

Give the personal details and the organisational affiliations of each member of the review team. Affiliation refers to groups or organisations to which review team members belong.
NOTE: email and country now MUST be entered for each person, unless you are amending a published record.

Dr Zufishan Alam. Centre for Health Services Research, Faculty of Medicine,The University of Queensland
Dr Joanne Cairns. Academy of Primary Care, Hull York Medical School, University of Hull

12. * Funding sources/sponsors.

Details of the individuals, organizations, groups, companies or other legal entities who have funded or sponsored the review.

The University of Queensland

Grant number(s)
State the funder, grant or award number and the date of award

13. * Conflicts of interest.

List actual or perceived conflicts of interest (financial or academic).

None

14. Collaborators.

Give the name and affiliation of any individuals or organisations who are working on the review but who are not listed as review team members. **NOTE: email and country must be completed for each person, unless you are amending a published record.**

15. * Review question. [1 change]

State the review question(s) clearly and precisely. It may be appropriate to break very broad questions down into a series of related more specific questions. Questions may be framed or refined using PI(E)COS or similar where relevant.

What is the effect of interventions on cervical screening uptake among migrant women?

16. * Searches. [1 change]

State the sources that will be searched (e.g. Medline). Give the search dates, and any restrictions (e.g. language or publication date). Do NOT enter the full search strategy (it may be provided as a link or attachment below.)

PubMed, Embase, Cumulated Index to Nursing and Allied Health Literature (CINAHL), PsycINFO, Scopus, The Cochrane Central Register of Controlled Trials (CENTRAL) and ERIC (Education Resources Information Centre) were searched from the inception of databases to 12th October 2021 for studies in English language. Handsearching of bibliography of included studies for any other relevant publications was done. The search terms to be used included 'cervical cancer' AND 'screening' AND 'migrant' AND 'refugee'. The titles and abstracts were then searched to include studies involving 'intervention' or 'health promotion'.

17. URL to search strategy. [1 change]

Upload a file with your search strategy, or an example of a search strategy for a specific database, (including the keywords) in pdf or word format. In doing so you are consenting to the file being made publicly accessible.

Or provide a URL or link to the strategy. Do NOT provide links to your search **results**.

https://www.crd.york.ac.uk/PROSPEROFILES/192341_STRATEGY_20211014.pdf

Do not make this file publicly available until the review is complete

18. * Condition or domain being studied. [1 change]

Give a short description of the disease, condition or healthcare domain being studied in your systematic review.

Cervical cancer is one of the leading causes of death among women all over the world. The burden of disease is predominantly carried by women in developing countries. Due to ineffective cervical screening programs in their home countries, migrant and refugee women may have insufficient knowledge about the importance of cervical screening, and therefore low screening attendance. In order to increase cervical screening uptake, interventions among migrant women are needed. A systematic review, summarizing literature on the interventions, carried out till now, focused on migrant and refugee women, is therefore essential to assess the effectiveness of conducted interventions.

19. * Participants/population. [1 change]

Specify the participants or populations being studied in the review. The preferred format includes details of both inclusion and exclusion criteria.

Migrant, immigrant or refugee women, settled in various countries of the world. Women of all ages will be included as cervical screening guidelines are different in different countries of the world.

20. * Intervention(s), exposure(s). [1 change]

Give full and clear descriptions or definitions of the interventions or the exposures to be reviewed. The preferred format includes details of both inclusion and exclusion criteria.

Cervical cancer screening interventions are defined as those applied to populations, groups or individuals with the purpose of changing personal behaviour to increase cervical cancer screening uptake. There are many approaches that can be used to attain this, including provision of cervical cancer information, training of General Practitioners and health care workers, use of mobile applications, facilitation in appointment scheduling and commutation, setting up of clinics and mailing self sampling test kits. These interventions have been broadly used for difficult to reach populations such as migrant women as well as socioeconomically disadvantaged people.

21. * Comparator(s)/control.

Where relevant, give details of the alternatives against which the intervention/exposure will be compared (e.g. another intervention or a non-exposed control group). The preferred format includes details of both inclusion and exclusion criteria.

Non exposed control group, usual care group or group provided with another form of intervention.

22. * Types of study to be included. [1 change]

Give details of the study designs (e.g. RCT) that are eligible for inclusion in the review. The preferred format includes both inclusion and exclusion criteria. If there are no restrictions on the types of study, this should be stated.

All forms of cervical cancer screening intervention studies i.e., randomised control trials, non randomised controlled trials as well as quasi controlled trials (pre-post studies) will be included in the review. These will include any sort of intervention (educational, behavioural etc) targeting increase in cervical screening uptake. Non English studies, those that do not contain intervention and are focused on determinants of cervical screening uptake, were not be included. The studies without main outcome measure (screening uptake) or those reporting qualitative findings were also excluded. The studies focusing on interventions designed for women after diagnosis of cervical cancer and rehabilitation were excluded. Studies on intervention of screening for conditions other than cervical cancer were excluded.

23. Context. [1 change]

Give summary details of the setting or other relevant characteristics, which help define the inclusion or exclusion criteria.

Research in all countries of the world where migrant women have settled were included. These could be based in the community, health care centres, places of worship, hospitals. no exclusion was done based on setting.

24. * Main outcome(s). [1 change]

Give the pre-specified main (most important) outcomes of the review, including details of how the outcome is defined and measured and when these measurement are made, if these are part of the review inclusion criteria.

Effect on cervical screening uptake on migrant women in the intervention group compared to control groups.
For meta-analysis (of randomized and non randomized controlled studies) post intervention difference in proportion of women who had had a cervical screening test in intervention and control groups.

Measures of effect

Effect on cervical screening uptake will be measured in terms of percentage difference between baseline and post intervention uptake of women that had had the test in both groups.
Risk ratio and risk difference will be used as measures of effect for meta-analysis.

25. * Additional outcome(s). [1 change]

List the pre-specified additional outcomes of the review, with a similar level of detail to that required for main outcomes. Where there are no additional outcomes please state 'None' or 'Not applicable' as appropriate to the review

Due to extensive scope of main outcomes, no additional outcomes will be measured.

Measures of effect

26. * Data extraction (selection and coding). [1 change]

Describe how studies will be selected for inclusion. State what data will be extracted or obtained. State how this will be done and recorded.

Study selection: Two independent reviewers imported the studies using the search strategy from databases to EndNote. Covidence was also used for review management. The titles and abstracts were screened to include relevant studies. Full text of the relevant publications were then assessed to include final studies. Any conflict, if faced, was be resolved by mutual discussion, or involvement of third person where required.
Data Extraction: This was done independently by both reviewers using a template with predesignated fields The fields included: publication date and authorship, setting/location, theoretical basis, sample size, recruitment, details on type and components of intervention and outcome measures. The extracted data was recorded in excel spreadsheet. For any missing data, the author of the study was contacted.

27. * Risk of bias (quality) assessment. [1 change]

State which characteristics of the studies will be assessed and/or any formal risk of bias/quality assessment tools that will be used.

Effective Public Healthcare Panacea Project (EPHPP) tool was used to assess risk of bias and quality of the included studies. EPHPP tool assess quality and risk of bias of the studies through key sections based on selection bias, study design, confounders, blinding, data collection methods, withdrawals and dropouts, intervention integrity and analysis appropriateness. No studies was excluded on the basis of risk of bias. Both reviewers conducted assessment independently. Disagreements were resolved by mutual discussion or via help of third person where relevant.

28. * Strategy for data synthesis. [1 change]

Describe the methods you plan to use to synthesise data. This **must not be generic text** but should be **specific to your review** and describe how the proposed approach will be applied to your data.

If meta-analysis is planned, describe the models to be used, methods to explore statistical heterogeneity, and software package to be used.

A narrative synthesis of findings structured around study setting, intervention characteristics and outcome measures will be provided from the included studies.

For the meta-analysis, a table based on quantitative findings from the extracted data, regarding intervention and treatment groups, will be prepared. It will be used to calculate risk ratios and risk differences. Due to diverse nature of interventions involved, we plan to conduct a random effects meta analysis.

29. * Analysis of subgroups or subsets. [1 change]

State any planned investigation of 'subgroups'. Be clear and specific about which type of study or participant will be included in each group or covariate investigated. State the planned analytic approach.

If enough studies are available subgroup meta-analysis for multi faceted versus simple interventions, source of outcome report, quality of the studies, self sampling versus non self sampling interventions, will be conducted

30. * Type and method of review. [1 change]

Select the type of review, review method and health area from the lists below.

Type of review

Cost effectiveness	No
Diagnostic	No
Epidemiologic	No
Individual patient data (IPD) meta-analysis	No
Intervention	Yes
Living systematic review	No
Meta-analysis	Yes
Methodology	No
Narrative synthesis	Yes
Network meta-analysis	No
Pre-clinical	No
Prevention	Yes
Prognostic	No
Prospective meta-analysis (PMA)	No
Review of reviews	No
Service delivery	No
Synthesis of qualitative studies	No
Systematic review	Yes
Other	No

Health area of the review

Alcohol/substance misuse/abuse	No
Blood and immune system	No
Cancer	Yes
Cardiovascular	No
Care of the elderly	No
Child health	No
Complementary therapies	No
COVID-19	No
Crime and justice	No
Dental	No
Digestive system	No
Ear, nose and throat	No
Education	No
Endocrine and metabolic disorders	No
Eye disorders	No
General interest	No
Genetics	No
Health inequalities/health equity	Yes
Infections and infestations	No
International development	No
Mental health and behavioural conditions	No
Musculoskeletal	No
Neurological	No
Nursing	No
Obstetrics and gynaecology	Yes
Oral health	No
Palliative care	No
Perioperative care	No
Physiotherapy	No
Pregnancy and childbirth	No
Public health (including social determinants of health)	Yes

Rehabilitation	No
Respiratory disorders	No
Service delivery	No
Skin disorders	No
Social care	No
Surgery	No
Tropical Medicine	No
Urological	No
Wounds, injuries and accidents	No
Violence and abuse	No

31. Language. [1 change]

Select each language individually to add it to the list below, use the bin icon to remove any added in error.

English

There is an English language summary.

32. * Country. [1 change]

Select the country in which the review is being carried out. For multi-national collaborations select all the countries involved.

Australia
England

33. Other registration details.

Name any other organisation where the systematic review title or protocol is registered (e.g. Campbell, or The Joanna Briggs Institute) together with any unique identification number assigned by them.
If extracted data will be stored and made available through a repository such as the Systematic Review Data Repository (SRDR), details and a link should be included here. If none, leave blank.

34. Reference and/or URL for published protocol.

If the protocol for this review is published provide details (authors, title and journal details, preferably in Vancouver format)

No I do not make this file publicly available until the review is complete

35. Dissemination plans. [1 change]

Do you intend to publish the review on completion?

Yes

The review will be published in a journal.

36. Keywords. [1 change]

Give words or phrases that best describe the review. Separate keywords with a semicolon or new line. Keywords help PROSPERO users find your review (keywords do not appear in the public record but are included in searches). Be as specific and precise as possible. Avoid acronyms and abbreviations unless these are in wide use.

cervical cancer; screening; immigrant; migrant; refugee; intervention

37. Details of any existing review of the same topic by the same authors.

If you are registering an update of an existing review give details of the earlier versions and include a full bibliographic reference, if available.

38. * Current review status.

Update review status when the review is completed and when it is published.
New registrations must be ongoing so this field is not editable for initial submission.

Review_Ongoing

39. Any additional information.

Provide any other information relevant to the registration of this review.

40. Details of final report/publication(s) or preprints if available.

Leave empty until publication details are available OR you have a link to a preprint (NOTE: this field is not editable for initial submission).
List authors, title and journal details preferably in Vancouver format.
