# Supplementary material for: Interventions to increase cervical screening uptake among immigrant women: A systematic review and meta-analysis
Source: PLoS One. 2023 Jun 2;18(6):e0281976. doi: 10.1371/journal.pone.0281976 (PMC10237485; doi:10.1371/journal.pone.0281976)
Supplement: S1 Fig — (DOCX) [file pone.0281976.s002.docx]

# S4 Figure: Forest plots showing sub-group analysis for cervical screening intervention studies included in the meta-analysis

**Figure S4a. Comparison of screening uptake post-intervention by study design**

**Figure S4b. Comparison of screening uptake post-intervention by intervention complexity**

**Figure S4c. Comparison of screening uptake post-intervention by format of educative material**

**Figure S4d. Comparison of screening uptake post-intervention by baseline screening status of participants**

**Figure. S4e. Comparison of screening uptake post-intervention by mode of intervention delivery**

**Figure S4f. Comparison of screening uptake post-intervention by involvement of community health workers (CHW)**

**Figure S4g. Comparison of screening uptake post-intervention by length of follow-up**

**Figure S4h. Comparison of screening uptake post-intervention by source of outcome**

**Figure S4i. Comparison of screening uptake post-intervention by type of intervention control group**

**Figure S4j. Comparison of screening uptake post-intervention by application of theoretical model**

**Figure S4k. Comparison of screening uptake post-intervention by study quality**
