# Supplementary material for: Interventions to increase cervical screening uptake among immigrant women: A systematic review and meta-analysis
Source: PLoS One. 2023 Jun 2;18(6):e0281976. doi: 10.1371/journal.pone.0281976 (PMC10237485; doi:10.1371/journal.pone.0281976)
Supplement: S2 Table — (DOCX) [file pone.0281976.s004.docx]

# S3 Table: Search strategy as used in different databases

**S3a. Search strategy from Pubmed**

|  | **Search terms** | **Results** |
| --- | --- | --- |
| **#1** | Search: ((((cervix[Title/Abstract]) OR (cervical[Title/Abstract])) AND (cancer[Title/Abstract]))) OR (neoplasm[Title/Abstract]) | **160,589** |
| **#2** | Search: uterine cervical neoplasms[MeSH Terms] | **78,878** |
| **#3** | Search: uterine cervical neoplasms[MeSH Terms] | **194,792** |
| **#4** | Search: (early diagnosis[MeSH Terms]) OR (early detection of cancer[MeSH Terms]) | **58,817** |
| **#5** | Search: (early diagnosis[Title/Abstract]) OR (early detection[Title/Abstract]) | **156,401** |
| **#6** | Search: mass screening[MeSH Terms] | **136,130** |
| **#7** | Search: screen*[Title/Abstract] | **840,882** |
| **#8** | Search: (mass screening[MeSH Terms]) OR (screen*[Title/Abstract]) | **882,907** |
| **#9** | Search: (((early diagnosis[MeSH Terms]) OR (early detection of cancer[MeSH Terms])) OR ((early diagnosis[Title/Abstract]) OR (early detection[Title/Abstract]))) OR ((mass screening[MeSH Terms]) OR (screen*[Title/Abstract])) | **1,030,180** |
| **#10** | Search: ((((((cervix[Title/Abstract]) OR (cervical[Title/Abstract])) AND (cancer[Title/Abstract]))) OR (neoplasm[Title/Abstract])) OR (uterine cervical neoplasms[MeSH Terms])) AND ((((early diagnosis[MeSH Terms]) OR (early detection of cancer[MeSH Terms])) OR ((early diagnosis[Title/Abstract]) OR (early detection[Title/Abstract]))) OR ((mass screening[MeSH Terms]) OR (screen*[Title/Abstract]))) | **27,311** |
| **#11** | Search: emigrants and immigrants[MeSH Terms] | **13,973** |
| **#12** | Search: (((immigrant*[Title/Abstract]) OR (migrant*[Title/Abstract])) OR (emigrant*[Title/Abstract])) OR (refugee*[Title/Abstract]) | **56,782** |
| **#13** | Search: ((emigrants and immigrants[MeSH Terms]) AND (emigrants and immigrants[MeSH Terms])) OR ((((immigrant*[Title/Abstract]) OR (migrant*[Title/Abstract])) OR (emigrant*[Title/Abstract])) OR (refugee*[Title/Abstract])) | **60,578** |
| **#14** | Search: (((((((cervix[Title/Abstract]) OR (cervical[Title/Abstract])) AND (cancer[Title/Abstract]))) OR (neoplasm[Title/Abstract])) OR (uterine cervical neoplasms[MeSH Terms])) AND ((((early diagnosis[MeSH Terms]) OR (early detection of cancer[MeSH Terms])) OR ((early diagnosis[Title/Abstract]) OR (early detection[Title/Abstract]))) OR ((mass screening[MeSH Terms]) OR (screen*[Title/Abstract])))) AND (((emigrants and immigrants[MeSH Terms]) AND (emigrants and immigrants[MeSH Terms])) OR ((((immigrant*[Title/Abstract]) OR (migrant*[Title/Abstract])) OR (emigrant*[Title/Abstract])) OR (refugee*[Title/Abstract]))) | **415** |
| **#15** | Search: (((((((cervix[Title/Abstract]) OR (cervical[Title/Abstract])) AND (cancer[Title/Abstract]))) OR (neoplasm[Title/Abstract])) OR (uterine cervical neoplasms[MeSH Terms])) AND ((((early diagnosis[MeSH Terms]) OR (early detection of cancer[MeSH Terms])) OR ((early diagnosis[Title/Abstract]) OR (early detection[Title/Abstract]))) OR ((mass screening[MeSH Terms]) OR (screen*[Title/Abstract])))) AND (((emigrants and immigrants[MeSH Terms]) AND (emigrants and immigrants[MeSH Terms])) OR ((((immigrant*[Title/Abstract]) OR (migrant*[Title/Abstract])) OR (emigrant*[Title/Abstract])) OR (refugee*[Title/Abstract]))) Filters: Full text | **392** |

**S3b. Search strategy from EMBASE**

|  | **Search strategy** | **Results** |
| --- | --- | --- |
| #1 | cervi* AND cancer | 176,897 |
| #2 | 'uterine cervix tumor'/exp/mj | 84,763 |
| #3 | 'uterine cervix cancer'/exp/mj | 66,735 |
| #4 | cancer AND of AND the AND cervix | 118,963 |
| #5 | #1 OR #2 OR #3 OR #4 | 190,760 |
| #6 | screen* | 1,501,000 |
| #7 | 'early cancer diagnosis' | 9,755 |
| #8 | early AND detect* | 407,811 |
| #9 | 'screening'/exp/mj OR 'early cancer diagnosis'/exp/mj | 213,809 |
| #10 | #6 OR #7 OR #8 OR #9 | 1,845,651 |
| #11 | #5 AND #10 | 38,571 |
| #12 | immigrant* OR migrant* OR refugee* | 73,768 |
| #13 | 'immigrant'/exp/mj OR 'refugee'/exp/mj | 13,911 |
| #14 | #12 OR #13 | 73,909 |
| #15 | #11 AND #14 | 539 |
| #16 | #15 AND ('papillomavirus infection'/dm OR' sexually transmitted disease'/dm OR 'uterine cervix cancer'/dm OR 'uterine cervix carcinoma'/dm OR 'uterine cervix carcinoma in situ'/dm OR 'uterine cervix disease'/dm OR 'uterine cervix tumor'/dm OR 'uterine cancer'/dm) | 480 |

**S3c. Search strategy from CENTRAL**

|  | **Search strategy** | **Results** |
| --- | --- | --- |
| #1 | (cervi* cancer) OR (cancer of the cervix) | 6737 |
| #2 | (immigrant*) OR (migrant*) OR (refugee*) | 1762 |
| #3 | (screen*) OR (early diagnosis) OR (early detect*) | 113922 |
| #4 | #1 and #3 | 1969 |
| #5 | #2 and #4 | 53 |

**S3d. Search strategy from CINAHL**

|  | **Search strategy** | **Results** |
| --- | --- | --- |
| S1 | TX cervical cancer | 15,607 |
| S2 | MH cervix neoplasms | 16,944 |
| S3 | S1 OR S2 | 22,406 |
| S4 | TX cervical cancer OR TX cervi* cancer OR TX cancer of the cervix | 16,350 |
| S5 | S3 OR S4 | 22,848 |
| S6 | MH early diagnosis | 11,507 |
| S7 | MH early detection of cancer | 10,568 |
| S8 | MH cancer screening | 15,869 |
| S9 | S6 OR S7 OR S8 | 37,000 |
| S10 | TX screen* OR TX early detect* | 264,688 |
| S11 | S9 OR S10 | 272,343 |
| S12 | MH immigrants | 15,701 |
| S13 | TX immigrant* OR TX migrant* OR TX refugee* | 37,912 |
| S14 | S12 OR S13 | 37,912 |
| S15 | S5 AND S11 | 9077 |
| S16 | S14 AND S15 | 356 |

**S3e. Search strategy from ERIC**

| S1 | cervi* cancer | 125 |
| --- | --- | --- |
| S2 | cancer of the cervix | 6 |
| S3 | (cervi* cancer) OR (cancer of the cervix) | 125 |
| S4 | screen* | 20,227 |
| S4 | early detect* | 1891 |
| S5 | (screen*) OR (Early detect*) | 21,842 |
| S6 | ((cervi* cancer) OR (cancer of the cervix) AND (screen*) OR (Early detect*)) | 79 |
| S7 | immigrant* OR migrant* OR refugee* | 25318 |
| S8 | (((cervi* cancer) OR (cancer of the cervix)) AND (screen*) OR (Early detect*))) AND (immigrant* OR migrant* OR refugee*) | 3 |

**S3f. Search strategy from SCOPUS**

|  | **Search strategy** | **Results** |
| --- | --- | --- |
| #1 | ( ( TITLE-ABS-KEY ( cervical AND cancer ) OR TITLE-ABS-KEY ( cervi* AND cancer ) OR TITLEABS-KEY ( cancer AND of AND the AND cervix ) ) ) AND ( ( TITLE-ABS-KEY ( screen* ) OR TITLE-ABS-KEY ( early AND detect* ) ) ) | 33766 |
| #2 | (TITLE-ABS-KEY ( immigrant* ) OR TITLE-ABS-KEY ( migrant* ) OR TITLE-ABS-KEY ( refugee* ) ) | 199,008 |
| #3 | ( ( ( TITLE-ABS-KEY ( cervical AND cancer ) OR TITLE-ABS-KEY ( cervi* AND cancer ) OR TITLE-ABS-KEY ( cancer AND of AND the AND cervix ) ) ) AND ( ( TITLE-ABS-KEY ( screen* ) OR TITLEABS-KEY ( early AND detect* ) ) ) ) AND ( ( TITLE-ABS-KEY ( immigrant* ) OR TITLE-ABS-KEY ( migrant* ) OR TITLE-ABS-KEY ( refugee* ) ) ) | 480 |

**S3g. Search strategy from PsycINFO**

|  | **Search strategy** | **Results** |
| --- | --- | --- |
| **1** | Index term: cervix | **1085** |
| **2** | Any field: cancer of the cervix | **943** |
| **3** | Any field: cervi* cancer | **3216** |
| **4** | ((Index term: cervix))) OR ((Any field: (cancer of the cervix))) OR ((Any field: (cervi* cancer))) | **3480** |
| **5** | Any field: screen* OR Any field: early detect* OR Any field: early diagnosis | **151909** |
| **6** | Index term: screening | **28179** |
| **7** | ((Index term: (screening))) OR ((Any field: (screen*)) OR (Any field: (early detect*)) OR (Any field: (early diagnosis)) | **151909** |
| **8** | ((((Index Terms: (screening)))) OR (((Any Field: (screen*))) OR ((Any Field: (early detect*))) OR ((Any Field: (early diagnosis))))) AND ((((Any Field: (cervi* cancer)))) OR (((Any Field: (cancer of the cervix)))) OR (((Index Terms: (cervix))))) | **1971** |
| **9** | Any field: immigrant* OR Any field: migrant* OR Any field: refugee* | **47868** |
| **10** | Index terms: immigrant OR Index terms: immigration | **24185** |
| **11** | ((Index Terms: (immigrants)) OR (Index Terms: (immigration))) OR ((Any Field: (immigrant*)) OR (Any Field: (migrant*)) OR (Any Field: (refugee*))) | **50446** |
| **12** | ((((Index Terms: (immigrants))) OR ((Index Terms: (immigration)))) OR (((Any Field: (immigrant*))) OR ((Any Field: (migrant*))) OR ((Any Field: (refugee*))))) AND ((((((Index Terms: (screening))))) OR ((((Any Field: (screen*)))) OR (((Any Field: (early detect*)))) OR (((Any Field: (early diagnosis)))))) AND (((((Any Field: (cervi* cancer))))) OR ((((Any Field: (cancer of the cervix))))) OR ((((Index Terms: (cervix))))))) | **140** |

**S3i: References retrieved from bibliography of relevant articles**

| 1 | McAvoy BR, Raza R. Can health education increase uptake of cervical smear testing among Asian women? *Br J Med*. 1991;302(6780):833-6. |
| --- | --- |
| 2 | Kernohan E. Evaluation of a pilot study for breast and cervical cancer screening with Bradford's minority ethnic women; a community development approach, 1991-93. *Br J Cancer Suppl*. 1996;29:S42 |
| 3 | Bird, J. A., McPhee, S. J., Ha, N. T., Le, B., Davis, T., & Jenkins, C. N. Opening pathways to cancer screening for Vietnamese-American women: lay health workers hold a key. *Preventive medicine*, 1998; *27*(6), 821-829. |
| 4 | Jibaja-Weiss ML, Volk RJ, Kingery P, Smith QW, Holcomb JD. Tailored messages for breast and cervical cancer screening of low-income and minority women using medical records data. *Patient Educ Couns*. 2003;50(2):123-32. |
| 5 | Han H-R, Song Y, Kim M, Hedlin HK, Kim K, Ben Lee H, et al. Breast and cervical cancer screening literacy among Korean American women: A community health worker–led intervention. *Am J Public Health*. 2017;107(1):159-65. |
| 6 | Kobetz E, Seay J, Koru-Sengul T, Bispo JB, Trevil D, Gonzalez M, et al. A randomized trial of mailed HPV self-sampling for cervical cancer screening among ethnic minority women in South Florida. C*ancer Causes Control*. 2018;29(9):793-801 |
| 7 | Fernandez ME, Lin J, Leong-Wu C, Aday L. Pap smear screening among Asian Pacific Islander women in a multisite community-based cancer screening program. *Health Promot Pract.*. 2009;10(2):210-21 |
| 8 | Chan D, So W. The impact of community-based multimedia intervention on the new and repeated cervical cancer screening participation among South Asian women. *Public Health.* 2020;178:1-4. |
| 9 | Wong CL, Choi KC, Chen J, Law BM, Chan DN, So WK. A community health worker–led multicomponent program to promote cervical cancer screening in South Asian women: a cluster RCT. American Journal of Preventive Medicine. 2021 Jul 1;61(1):136-45. |
| 10 | So WK, Kwong AN, Chen JM, Chan JC, Law BM, Sit JW, Chan CW. A theory-based and culturally aligned training program on breast and cervical cancer prevention for South Asian community health workers: a feasibility study. Cancer Nursing. 2019 Mar 1;42(2):E20-30. |
| 11 | Cullerton K, Gallegos D, Ashley E, Do H, Voloschenko A, Fleming M, Ramsey R, Gould T. Cancer screening education: can it change knowledge and attitudes among culturally and linguistically diverse communities in Queensland, Australia?. Health Promotion Journal of Australia. 2016 Jun 29;27(2):140-7. |
| 12 | White K, Garces IC, Bandura L, McGuire AA, Scarinci IC. Design and evaluation of a theory-based, culturally relevant outreach model for breast and cervical cancer screening for Latina immigrants. Ethnicity & disease. 2012;22(3):274. |
| 13 | Nuño T, Martinez ME, Harris R, García F. A promotora-administered group education intervention to promote breast and cervical cancer screening in a rural community along the US–Mexico border: a randomized controlled trial. Cancer Causes & Control. 2011 Mar;22(3):367-74. |
| 14 | Tosomeen AH, Marquez MA, Panser LA, Kottke TE. Developing preventive health programs for recent immigrants: a case study of cancer screening for Vietnamese women in Olmsted County, Minnesota. Minnesota medicine. 1996 May;79(5):46-8. |
| 15 | Hyman I, Cameron JI, Singh PM, Stewart DE. Physicians and pap testing in the Chinese and Vietnamese communities in Toronto. Journal of Health Care for the Poor and Underserved. 2003;14(4):489-502. |
| 16 | Erwin DO, Johnson VA, Feliciano-Libid L, Zamora D, Jandorf L. Incorporating cultural constructs and demographic diversity in the research and development of a Latina breast and cervical cancer education program. Journal of Cancer Education. 2005 Mar 1;20(1):39-44. |
| 17 | Smith D, Hodgson SR, Schwartz M, Gorman B, Mody D, Chevez-Barrios P, Coffey D. Cervical Cancer Screening in a Never or Rarely Screened Indigent Inner City Latina Population: A Collaborative Project of the CAP See, Test, and Treat Program, Diaz de la Mujer Latina and The Methodist Hospital. Journal of the American Society of Cytopathology. 2012;1(1):S54. |
