# Supplementary material for: Interventions to increase cervical screening uptake among immigrant women: A systematic review and meta-analysis
Source: PLoS One. 2023 Jun 2;18(6):e0281976. doi: 10.1371/journal.pone.0281976 (PMC10237485; doi:10.1371/journal.pone.0281976)
Supplement: S3 Table — (DOCX) [file pone.0281976.s005.docx]

# S5 Table: Quality appraisal of studies included in the systematic review

|  | **Quality Appraisal Criteria** | | | | | | |
| --- | --- | --- | --- | --- | --- | --- | --- |
| **Study** | **Overall Study quality** | **Selection bias** | **Study design** | **Confounders** | **Blinding** | **Data collection** | **Withdrawals & dropouts** |
| Bird et al., 1998 | Strong | Moderate | Strong | Strong | Moderate | Strong | Moderate |
| Black et al., 2006 | Weak | Weak | Moderate | Weak | Moderate | Weak | Weak |
| Brown et al., 2018 | Weak | Weak | Moderate | Weak | Moderate | Strong | Strong |
| Byrd et al., 2013 | Weak | Weak | Strong | Weak | Moderate | Moderate | Strong |
| Carrasquillo et al., 2018 | Moderate | Weak | Strong | Strong | Moderate | Strong | Strong |
| Chan et l., 2019 | Weak | Moderate | Strong | Weak | Weak | Weak | Weak |
| Choy et al., 2021 | Moderate | Weak | Moderate | Strong | Moderate | Weak | Strong |
| Dietrich et al., 2006 | Strong | Moderate | Strong | Strong | Moderate | Moderate | Strong |
| Dunn et al., 2017 | Weak | Weak | Moderate | Moderate | Moderate | Strong | Weak |
| Elder et al., 2016 | Moderate | Weak | Strong | Weak | Strong | Moderate | Strong |
| Fernandez et al., 2009 | Weak | Weak | Weak | Weak | Moderate | Weak | Weak |
| Fernandez et al., 2020 | Moderate | Moderate | Moderate | Weak | Moderate | Moderate | Strong |
| Goldsmith et al., 1996 | Weak | Moderate | Moderate | Weak | Moderate | Strong | Weak |
| Grewal et al., 2004 | Weak | Weak | Weak | Weak | Moderate | Weak | Weak |
| Han et al., 2015 | Moderate | Weak | Moderate | Strong | Moderate | Strong | Strong |
| Ilangovan et al., 2016 | Weak | Weak | Weak | Weak | Moderate | Strong | Strong |
| Jackson et al., 2002 | Strong | Moderate | Strong | Strong | Moderate | Moderate | Strong |
| Jandorf et al., 2014 | Moderate | Weak | Strong | Weak | Moderate | Weak | Weak |
| Jenkins et al., 1999 | Moderate | Weak | Strong | Strong | Moderate | Strong | Strong |
| Jibaja et al., 2003 | Moderate | Moderate | Strong | Weak | Moderate | Strong | Strong |
| Kernohan at al., 1996 | Weak | Moderate | Weak | Weak | Moderate | Weak | Moderate |
| Kiser et al., 2020 | Weak | Weak | Weak | Weak | Moderate | Strong | Strong |
| Kobetz et al., 2018 | Moderate | Weak | Strong | Strong | Moderate | Moderate | Moderate |
| Lam et al., 2003 | Weak | Weak | Strong | Weak | Moderate | Moderate | Strong |
| Luque et al., 2017 | Moderate | Weak | Moderate | Strong | Moderate | Moderate | Strong |
| Ma et al., 2015 | Moderate | Weak | Strong | Weak | Moderate | Strong | Strong |
| Maxwell et al., 2003 | Moderate | Weak | Strong | Strong | Moderate | Strong | Moderate |
| McAvoy et al., 1991 | Weak | Moderate | Moderate | Moderate | Moderate | Weak | Weak |
| Meade et al., 2002 | Weak | Weak | Moderate | Weak | Moderate | Strong | Weak |
| Moen et al., 2020 | Moderate | Moderate | Strong | Strong | Moderate | Strong | Weak |
| Nuno et al., 2011 | Strong | Strong | Strong | Strong | Moderate | Strong | Strong |
| O’Brien et al., 2010 | Weak | Weak | Strong | Strong | Moderate | Strong | Weak |
| Ochua et al ., 2020 | Weak | Moderate | Strong | Strong | Moderate | Weak | Weak |
| Qureshi et al., 2021 | Weak | Weak | Strong | Strong | Moderate | Moderate | Weak |
| Savas et al., 2018 | Weak | Weak | Strong | Strong | Weak | Weak | Strong |
| Sewali et al., 2015 | Moderate | Weak | Strong | Strong | Moderate | Moderate | Strong |
| Taylor et al., 2002 | Strong | Strong | Strong | Strong | Moderate | Strong | Moderate |
| Wang et al., 2010 | Weak | Weak | Moderate | Moderate | Moderate | Strong | Weak |
| White et al., 2012 | Weak | Weak | Weak | Weak | Moderate | Weak | Weak |
| Wong et al., 2008 | Weak | Weak | Moderate | Weak | Moderate | Weak | Strong |
| Wong et al., 2019 | Moderate | Moderate | Strong | Weak | Moderate | Strong | Strong |
| Wong et al., 2021 | Moderate | Moderate | Strong | Strong | Moderate | Strong | Strong |
