# Supplementary material for: Interventions to increase cervical screening uptake among immigrant women: A systematic review and meta-analysis
Source: PLoS One. 2023 Jun 2;18(6):e0281976. doi: 10.1371/journal.pone.0281976 (PMC10237485; doi:10.1371/journal.pone.0281976)
Supplement: Attachment — Submitted filename: Response to reviewers.docx [file pone.0281976.s006.docx]

**Response to reviewers**

We are grateful to both the reviewers for providing us feedback and opportunity to improve the manuscript. We have endeavoured to revise the manuscript thoroughly in line with the suggestions. We have provided detailed explanation to each comment raised by the reviewer, the response is given (in blue) and changes/additions in the manuscript text (in red).

**Reviewer #1**: This is a very well presented work, and it is methodologically correct. It is easy to follow and the conclusions are appropriate. From my point of view, it meets the criteria to be published. However, I would like to raise some issues or corrections to the authors.
1. On page 4, line 88, it is commented that the references of the included articles were revised. It would be good if the results were commented on which result this search brought. I think a point is made only in the discussion.

The references for studies obtained from bibliography of relevant articles have been added as an additional supplemental table that has been referenced in the text to show the results brought by this research, as follows:

Additionally, bibliographies of included articles were hand-searched to identify other potentially relevant studies (S3 table i) (Page 5, Line 111-112)

2. In table 1, the sum should always be 42. Could you please check if this is an error or if this is data not available? In this case, I recommend adding it to the table.

Thank you for bringing this to our attention, the number of studies in each category in Table 1 has been revised accordingly.

3. On page 9, line 183, specific subgroups workers are discussed, but it is not clear to which other groups the rest belong and whether the data is in the table or not

Other groups refer to general women in community, not belonging to any specific profession. Table 2 gives the information on ethnicity, and specifies if any special group was present (Nail salon workers, farmworkers and migrant sex workers). Further elaboration has been provided in text as follows:

Most of the studies involved immigrant women from the community not belonging to any specific profession, while six studies focused on specific subgroups i.e., nail salon workers, farmworkers or female sex workers (Table 2) (Page 10, Lines: 213-2015)

4. The authors show a heterogeneity index of 93%, and add: "indicating considerable heterogeneity". From my point of view, it would be a matter of high heterogeneity. In any case, it would be better not to make any assessment in the results section and deepen the impact of the same on the possible conclusions.

We agree with your comment, the text in results has been revised to replace considerable with high (line 43). Conclusion has been revised to add:

However, due to substantial heterogeneity observed in the meta-analysis results, intervention effects need to be interpreted cautiously. (Page 32, Lines: 422-424)

5. On page 28, first sentence, the authors comment about a Kappa index of 0.4. Again, I recommend to comment on this in the discussion section.

Comment regarding Kappa index has been added in the discussion section as follows:

Additionally, low Kappa score as evaluated in our study indicates weak interrater reliability on the quality appraisal of the studies. However, the literature suggests that the Kappa index may amplify disagreement estimate among the raters [64]. A low Kappa index is more of a concern when dealing with diagnostic tests in clinical studies [65] compared to quality appraisal as in the current study. (Page 29, Lines: 336-340)

6. In the discussion, line??, the authors comment that the meta-analyses suggested a moderate ES of 1.15 (95% CI 1.03-1.29). I consider that it could be considered a low ES, taking into account that the lower limit is 1.03, and the upper one is 1.29.

Agreed, we have replaced ‘moderate’ with ‘low’ ES. (Page 29, Line 327)

7. Finally, I suggest the authors to include some discussion or consideration about the high cost-benefit of the interventions, given the small benefit shown by the pooled analyses, and the type of intervention (targeted at specific groups) that might seem more effective. How much money to save one woman from death from cancer de cervix, or how much to detect one cervical cancer?

We are grateful for bringing to our attention this point, it has been added as suggested in the discussion as follows:

None of the included studies reported cost-effectiveness of the intervention used, it is recommended to include this outcome in future studies. This would be of high practical value, allowing the policymakers to understand the value of the choice of intervention, including of HPV self-sampling method. A recent systematic review of studies assessing cost of HPV self-sampling compared to standard screening strategies, reported it to be highly cost -effective for under-screened women in high income countries, either when offered alone or in combination with other strategies. (Page 30, Lines 391-397)

8. There seems to be a mistake in reference 67. Could you please check?

Reference has been revised as follows:

Eccles M. The Improved Clinical Effectiveness Through Behavioral Research Group (ICEBERG). Designing theoretically-informed implementation interventions. Implementation Science. 2006;23;1(1):4.

**Reviewer #2**: 1. Generally, I think the Introduction section is written well. However, it would be better if the authors can strengthen the following, as well as in Discussion: (1) compared to previous “individual” studies (i.e., not a meta-analysis level study) in this topic, what is the new value from the meta-analysis would be expected to bring into? (2) why executing this meta-analysis is so crucial for this topic? The considerations should greatly beyond “None of these previous reviews quantified the extent of benefit through meta-analysis” (page 3, lines 67-68); and (3) what are the questions this meta-analysis can further address but have not been answered in the past?

We thank reviewer two for the suggestion on further elaboration on the objectives of the meta- analysis. Introduction and discussion sections have been revised to include the suggested description:

**Introduction**

Three systematic reviews have summarised studies involving health promotion interventions to increase cervical screening uptake among at-risk population subgroups. Of those, two focused on specific migrant groups i.e., Asian and Hispanic immigrant populations and outlined the significance of of sociocultural factors and population characteristics in intervention effectiveness [7,8]. Whereas the third review of studies conducted between 2006-16 focused on activities for increasing cervical screening uptake among low socioeconomic groups indicating effectiveness of HPV self-sampling [9]. Reviews have been carried out to summarize the evidence on interventions that used specific strategies such as education provision, Human Papilloma Virus (HPV) self-sampling or health care provider (HCP) counselling, among the Indigenous/native women [10-12]. However, none of these previous reviews addressed the overall diverse immigrant populations in different parts of the world, nor summarized various intervention strategies for increasing cervical screening in immigrants. Given the recent launch of global initiative to eliminate cervical cancer as a public health problem by WHO [13], it is critical to systematically review the evidence on effectiveness of interventions, among under reached groups such as immigrants.

Thus, the objective of this study was to obtain the systematic evidence, expanding on immigrant population subgroups from various backgrounds, not limited to intervention strategies of specific type, as opposed to previous reviews and to compare the effect of intervention between intervention and control groups through meta-analysis. This review aimed to systematically summarise the global and up to date evidence on interventions aiming to increase cervical screening uptake among immigrant and refugee women, and quantify their effectiveness via providing a pooled estimate of the effect, through a meta-analysis. (Page 3, Lines 60-63, Page 4, Lines 70-72)

**Discussion**

Therefore, to our knowledge this is the first systematic review and meta-analysis to comprehensively map the global evidence on the effectiveness of interventions to increase cervical screening in immigrant women. It compared the intervention effect sizes based on characteristics such as delivery format, involvement of HCPs, forms of educative material, screening status of participants and type of control group. (Page 30, Lines 410-415)

2. The authors mentioned “Sensitivity analysis was also conducted…” (page 6, lines 139-140). I would suggest indicating clearly here about what a study with low quality exactly was (e.g., giving clear definition and calculation on quality score, an instance as well).

Further explanation has been included as follows in the methods section whweras the example of study has been given in results section:

Sensitivity analysis was also conducted by removing studies with low quality (that scored weak on EPHPP scale) as well as an evident outlier with the highest effect size. (Page 7, Lines 166-167, Methods section)

When sensitivity analysis was conducted by removing studies with low quality [26,61],, or those evident as outliers in the funnel plot [45], heterogeneity indices remained the same, and no effect was observed on the effect size. (Page 26, Line 306, Results section)

3. In Table 1 (page 7), why for the characteristic “Complexity of intervention”, the total number is not 42 instead of 41 (8+33)?

The number has been revised and corrected.

4. In Table 1 (page 8), would it be better if adjusting “Country” to “Location”? It seems that not all candidates are countries.
We agree, term ‘country’ has been replaced with ‘location’.

5. Followed by the previous one, please comment/judge that would it be an issue/effect on the meta-analysis result if pooling studies altogether but their location units were not the same (e.g., some from country level and others from city level)?
Further analysis of studies conducted at multiple locations (country level) and single location (city level), has been included. Results are displayed in Table 3 and explanation in text as follows:

Theoretically-guided intervention studies had higher ES, as did the ones involving community health workers and those conducted at broader level involving multiple locations within the country (Page 26, Lines 302-303)

6. Can the authors add some descriptions ahead (e.g., in the Method section) or somewhere appropriate about what the characteristic “Theoretically guided intervention” exactly indicate?

The detail has been added in the methods section as:

… guidance by a theoretical or behaviour change model (theoretically guided) (Page 6, Lines 144-145)

7. Would there be any reason that the characteristic “Date of publication” divide into the three categories? It would be better for readers if clarifying and adding this on the manuscript.
There was no specific purpose, the date of publication was evenly distributed according to decades i.e., 1990-2000, 2001-2010, 2011-2021

8. In Discussion, please give a more deep discussion on the following: (1) future research implication and direction based on this meta-analysis; and (2) relevant screening policies implications for immigrants.

Future research implication and direction, as suggested by Reviewer 1 also, has been added as follows:

None of the included studies reported cost-effectiveness of the intervention used, it is recommended to include this outcome in future studies. This would be of high practical value, allowing the policymakers to understand the value of the choice of intervention, including of HPV self-sampling method. A recent systematic review of studies assessing cost of HPV self-sampling compared to standard screening strategies, reported it to be highly cost -effective for under-screened women in high income countries, either when offered alone or in combination with other strategies[73] . Furthermore, effect of certain variables of interest, such as age and length of stay of participants in the country, could be explored through meta-regression in future studies. (Page 32, Lines 391-399)

Implications regarding relevant screening policies has been described as:

Therefore, policies designed to address cervical screening services and interventions for migrants should take into consideration, relevance of cultural responsiveness when including components such as information provision, navigation as well as financial incentives. (Page 29, Lines: 382-385).
