# Supplementary material for: Interventions to increase cervical screening uptake among immigrant women: A systematic review and meta-analysis
Source: PLoS One. 2023 Jun 2;18(6):e0281976. doi: 10.1371/journal.pone.0281976 (PMC10237485; doi:10.1371/journal.pone.0281976)
Supplement: Attachment — Submitted filename: response to reviewers.docx [file pone.0281976.s007.docx]

**Response to reviewers**

We are grateful to reviewer 3 for providing us feedback on the manuscript. We have addressed the comments and revised the manuscript as suggested. The detailed explanation to each comment raised by the reviewer is provided, the response is given (in blue) and changes/additions in the manuscript text (in red).

Reviewer #3: I read with great interest the Manuscript titled "Interventions to increase cervical screening uptake among immigrant women: a systematic review and meta-analysis " which falls within the aim of the Journal.
In my honest opinion, the topic is interesting enough to attract the readers’ attention. Methodology is accurate and conclusions are supported by the data analysis. Nevertheless, authors should clarify some point and improve the discussion citing relevant and novel key articles about the topic.

-The abstract should be improved (in particular the section on materials and methods) in order to better summarize the contents of the manuscript.

Abstract has been revised to include more details based on contents of the manuscript, keeping in view the abstract world limit. (*Page 2, lines 19-44*)

Numerous intervention studies have attempted to increase cervical screening uptake among immigrant women, nonetheless their screening participation remains low. This systematic review and meta-analysis aimed to summarise the evidence on interventions to improve cervical screening among immigrant women globally and identify their effectiveness. Databases PubMed, EMBASE, Scopus, PsycINFO, ERIC, CINAHL and CENTRAL were systematically searched from inception to October 12, 2021, for intervention studies, including randomised and clinical controlled trials (RCT, CCT) and one and two group pre-post studies. Peer-reviewed studies involving immigrant and refugee women, in community and clinical settings, were eligible. Comparator interventions were usual or minimal care or attention control. Data extraction, quality appraisal and risk of bias were assessed by two authors independently using COVIDENCE software. Narrative synthesis of findings was carried out, with the main outcome measure defined as the cervical screening uptake rate difference pre- and post-intervention followed by random effects meta-analysis of trials and two group pre-post studies, using Comprehensive Meta-Analysis software, to calculate pooled rate ratios and adjustment for publication bias, where found. The protocol followed PRISMA guidelines and was registered prospectively with PROSPERO (CRD42020192341). 1,900 studies were identified, of which 42 (21 RCTS, 4 CCTs, and 16 pre-post studies) with 44,224 participants, were included in the systematic review, and 28 with 35,495 participants in the meta-analysis. Overall, the uptake difference rate for interventions ranged from -6.7 to 96%. Meta-analysis demonstrated a pooled rate ratio of 1.15 (95% CI 1.03-1.29), with high heterogeneity. Culturally sensitive, multicomponent interventions, using different modes of information delivery and self-sampling modality were most promising. Interventions led to at least 15% increase in cervical screening participation among immigrant women. Interventions designed to overcome logistical barriers and use multiple channels to communicate culturally appropriate health promotion messages are most effective at achieving cervical screening uptake among immigrant women.

-The introduction should be extended and completed. I find interesting a reference to the efforts made for the prevention and early diagnosis of gynecological cancers (see PMID: 36141217).

Introduction has been revised, with addition of the suggested reference as follows (*Page 3, Lines 48-50*):

Research evidence clearly shows that secondary prevention in terms of screening can effectively reduce cervical cancer mortality [5]. Screening options now being employed worldwide include Pap and HPV test [6].

6. Giannini A, Bogani G, Vizza E, Chiantera V, Laganà AS, Muzii L, Salerno MG, Caserta D, D’Oria O. Advances on Prevention and Screening of Gynecologic Tumors: Are We Stepping Forward?. InHealthcare 2022 Aug 24 (Vol. 10, No. 9, p. 1605). MDPI.

- I also suggest authors to better organize the discussion section following this ideal structure: main findings of the study, strength and limitations of the study, implications and comparison with literature, future directions.

The discussion has been re organised following the suggested structure. The strengths and limitations have been moved before discussing implications and comparison with literature as follows (Page 27, Lines 296-307):

This study critically reviewed and meta-analysed interventions to increase cervical screening uptake among immigrant women globally. The review found that culturally appropriate interventions such as those providing HPV self-sampling methodology and targeted clinics for immigrants are most effective. Meta-analysis found that multicomponent interventions were more beneficial than single component ones, as were those theoretically guided, delivered in-person and using multiple formats of information delivery. Participant characteristics, such as baseline screening status also influenced the success of the interventions, as did the type of intervention selected for the control group.

To our knowledge this is the first systematic review and meta-analysis to comprehensively map the global evidence on the effectiveness of interventions to increase cervical screening in immigrant women. It compared the intervention effect sizes based on characteristics such as delivery format, involvement of HCPs, modes of educative material, screening status of participants and type of control group. The strengths of this study include following a prospectively registered protocol, clearly and transparently outlining our search strategy and methods of analysis, having multiple reviewers independently working on each review stage, and investigating novel methods of encouraging screening i.e., self-sampling, not included in previous intervention reviews on immigrants. However, this review also has certain limitations. First, although we conducted a comprehensive search of multiple databases, some studies might not have been located. We tried to overcome this through hand citation searches. Second, we found the EPHPP tool was more favorable towards experimental studies compared to non-controlled studies resulting in most studies being scored of weak quality. We also found significant heterogeneity in the studies limiting the number we could include in the sub-group meta-analyses.

The meta-analysis results suggested a low overall ES of 1.15 (95% CI 1.03–1.29) across all intervention studies after adjustment for publication bias…….

- Discussions can be expanded and improved by citing relevant articles (I suggest authors to read and insert in references the following article PMID: 35742340).
Considered all these points, I think it could be of interest for the readers and, in my opinion, it deserves the priority to be published after minor revisions.

We feel that the suggested reference fits better in the introduction section due to its focus on treatment of early and late stages of cervical cancer, rather than in the discussion. Therefore, we have included it in the introduction as follows (*Page 3, lines 50-51*):

Advances in biomedical research has led to introduction of novel surgical, radiotherapeutic and systemic options for the treatment of cervical cancer [4].

4. D’Oria O, Corrado G, Laganà AS, Chiantera V, Vizza E, Giannini A. New Advances in Cervical Cancer: From Bench to Bedside. International Journal of Environmental Research and Public Health. 2022 Jun 9;19(12):7094.
